# Quantum state holography to reconstruct the molecular wave packet using an attosecond XUV–XUV pump-probe technique

**DOI:** 10.1038/s41598-020-69733-1

**Published:** 2020-07-31

**Authors:** Alberto González-Castrillo, Fernando Martín, Alicia Palacios

**Affiliations:** 1 Instituto Madrileño de Estudios Avanzados (IMDEA) en Nanociencia, 28049 Madrid, Spain; 2 Departamento de Química, Modulo 13, Universidad Autónoma de Madrid, 28049 Madrid, Spain; 3 Condensed Matter Physics Center (IFIMAC), Universidad Autónoma de Madrid, 28049 Madrid, Spain; 4 Institute of Advanced Research in Chemical Sciences (IAdChem), Universidad Autónoma de Madrid, 28049 Madrid, Spain

**Keywords:** Attosecond science, Atomic and molecular interactions with photons

## Abstract

An attosecond molecular interferometer is proposed by using a XUV–XUV pump-probe scheme. The interferograms resulting in the photoelectron distributions enable the full reconstruction of the molecular wave packet associated to excited states using a quantum state holographic approach that, to our knowledge, has only been proposed for simple atomic targets combining attosecond XUV pulses with IR light. In contrast with existing works, we investigate schemes where one- and two-photon absorption paths contribute to ionize the hydrogen molecule and show that it is possible to retrieve the excitation dynamics even when imprinted in a minority channel. Furthermore, we provide a systematic analysis of the time-frequency maps that reveal the distinct, but tightly coupled, motion of electrons and nuclei.

## Introduction

Interferometry is a successfully employed technique in Optics as a tool to access the relative phases and amplitudes of interfering waves, widely extended to examine matter waves. In recent applications, interferometric techniques have demonstrated its suitability to obtain structural and dynamical information in atoms and molecules^1^ in experiments measuring the photoelectron spectrum as phase-sensitive observable. An example par excellence are the two-center interferences resulting in the ionization of a diatomic homonuclear molecule described by Cohen and Fano^2^, where the basic notions of the Young double slit optical experiment are applied. Several experiments using synchrotron radiation have exploited double-slit type approaches as a tool to capture the wave nature of heavy particles^3^ or the electron entanglement in bound electronic states in atoms^4^ or simple molecules^5^.

**Figure 1:**
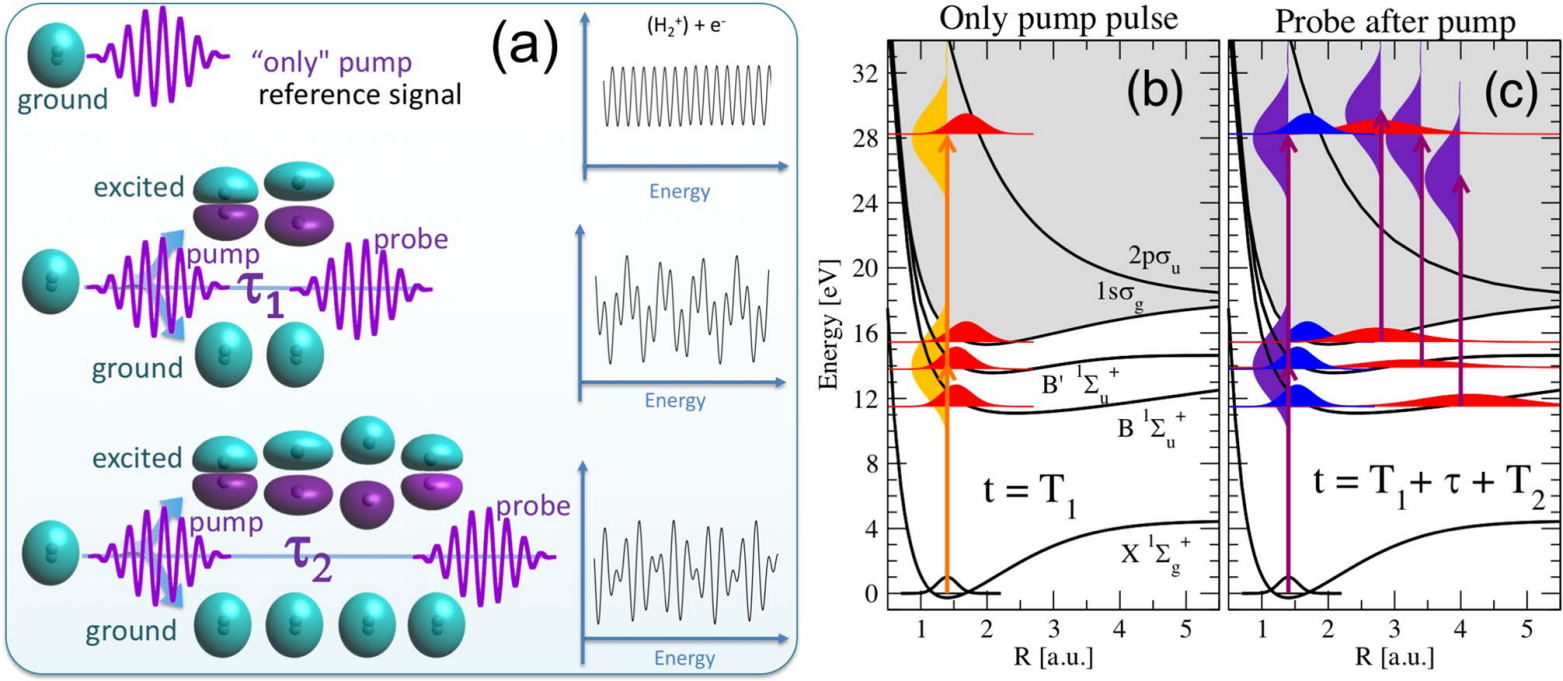
(**a**) Sketch of a molecular interferometer: we use as reference the ionization signal retrieved upon interaction with a single pulse. This interaction leads to ionization, but it also excites the molecule. The excitation dynamics however can only be captured by introducing a second pulse (probe). A series of experiments introducing a second pulse with different time delays ($\tau _1$, $\tau _2,\ldots$) with respect to the first one is then performed. The probe pulse can ionize from the ground state or from the coherently populated excited states. Optical and quantum interferences are then imprinted in the measurable ionization signal. The electron wave packet created upon ionization by the pump pulse alone (i.e., our reference wave) interfere with the one resulting after the interaction with the probe, thus imprinting the information of the excitation dynamics in our signal. (**b**) Schematic representation of the energetics and the attosecond pump-probe scheme using 2-fs pulses with a carrier frequency of 14 eV. The interaction with the pump pulse creates a wave packet with components in the ground, excited (through 1-photon absorption) and ionized molecule (through 1 and 2-photon absorption processes). We include the realistic distributions of the nuclear wave packet components for pump and probe pulses with 2-fs duration, $T_1=T_2=2$ fs, and a time delay $\tau$ between them of 6 fs. (**c**) The interaction with the probe pulse creates a replica of the wave packet generated by the identical pump pulse, but also induces ionization from the evolving wave packet in the excited molecule.

The advent of nano- and femto-second light pulses in the second half of the twentieth century led to novel approaches using the coherent signal of two accessible quantum paths to trace light-induced ultrafast nuclear dynamics^6–8^. The succeeding production of attosecond pulses by means of high-order harmonic generation (HHG) techniques then gave access to a real-time track and manipulation of electron wave packets, mainly by employing simple atoms as targets^9–15^. The temporal coherence of the ultrashort pulses is imprinted into the electronic wave packet, thus the resulting photoelectron spectra can be used to characterize the pulses^16–18^ or, alternatively, well-characterized pulses can be employed to retrieve amplitude-phase information from the electronic wave packet. Photoelectron interferometric techniques have also been recently proposed to map the ultrafast dynamics dictated by electronic wave packets associated to bound states^9–12,15^ or continuum states, with an unbound electron, as in the so-called RABBITT technique (Reconstruction of attosecond beating by interference of two-photon transitions) or attosecond streaking techniques^18^. RABBITT experiments have been mostly performed in atoms^19–21^ and, more recently, in small molecules^22–24^. All these experiments combine an attosecond XUV pulse (or a train of them) with a time-delayed IR femtosecond pulse. These approaches have provided rich dynamical information on atoms, such as the energy and angular dependencies of photoionization time delays, for which investigations using the simplest target with electron correlation, namely the He atom, have been crucial^11,12,14,15,19–21^. The basic common principle in all of them is the indirect measurement of the accumulated phase difference between distinct two-photon paths into the photoelectron yields. And the main issue to address is to establish a reference for the phase retrieval for an unambiguous determination of the wave function phase. For molecular targets, identifying the relevant interfering paths is a more difficult task, because of the multichannel character of the scattering wave function. The generation of attosecond molecular wave packets implies the description of coherently populated vibronic (electronic+vibrational) states, entering in a more complex picture than that of a disentangled analysis of individual nuclear wave packets^6–8^. Here we present an interferometric approach to obtain a full characterization of a molecular wave packet with attosecond resolution. We employ a scheme similar to that used in previous studies^25,26^, which make use of twin ultrashort XUV pulses, thus circumventing most of above-mentioned difficulties intrinsic to XUV–IR schemes. The hydrogen molecule is our testbed target as it allows for an exact theoretical description of the molecular dynamics. We will use a multi-path interference scheme shown in Fig. 1.

Our goal is to demonstrate how to attain an unambiguous full reconstruction of the XUV-induced dynamics of an excited molecule with attosecond resolution using a XUV–XUV pump-probe technique. In previous works, we have performed analogous studies^25,26^ by mapping the excitation dynamics into the dissociative ionization channel (H + H$^+$ + e$^-$). We have also investigated^26^ this problem by looking at the photoelectron spectrum resolved in both energy and angle (i.e. the molecular frame photoelectron angular distributions). Experimentally, this requires the availability of momentum-coincidence detection devices^27–30^. Moreover, one of the major challenges for the experimental implementation of this scheme are the still rather low peak intensities and repetition rates of attosecond pulses produced by HHG. These limitations could be overcome employing free electron lasers^31,32^ as they have recently broken the fs barrier, although in the X-ray frequency range^33^. Also, there still remains the issue of the shot-to-shot reproducibility of the pulses, which is a prerequisite for the investigation of coherent electron dynamics. For these reasons and in order to facilitate the retrieval of the ultrafast molecular dynamics by using HHG pulses, we focus on the information encoded in the total ionization yields or energy differential photoelectron yields, thus compensating the lack of intensity that is required to obtain channel- and angle-resolved information^34,35^.

In previous work^25,26^, we disentangled the interferometric patterns analyzing the ionization probabilities exclusively associated to two-photon absorption. In contrast, in the present work, we address the more realistic scenario where one-photon ionization also contributes. We demonstrate that, even though one-photon ionization largely dominates the photoelectron spectrum, it is still possible to unambiguously retrieve the dynamical information encoded in the minority two-photon contribution. More interestingly, we show that this information is encoded in the total yields, so that there is no need to look at angular and energy differential yields as in Ref.^26^, which would require momentum-coincidence measurements, thus increasing the complexity of an experimental realization. We show how the time-frequency maps of the total ionization yields reveal the distinct, but entangled, motion of electrons and nuclei, and that the photoelectron (or photoion) interferograms allow us for a full reconstruction of the molecular wave packet using a ”quantum state holographic” approach, following the same principles employed in experiments in helium atom^12,15^.

## Results

### Molecular interferometer

The hydrogen molecule, initially in its ground state ($\hbox {X}^1\Sigma _g^+,v=0$), is irradiated with two CEP-stabilized time-delayed attosecond XUV pulses. We employ collinear pulses with the light polarization along the molecular axis, which significantly reduces the size of the problem. The pulses have a total duration of 2 fs each and peak intensities of $10^{12}\, \hbox {W/cm}^2$, which can be nowadays experimentally realized and characterized in laboratories employing HHG techniques^35,36^. The time and energy distributions of the pairs of pulses are illustrated in the supplementary information. We choose carrier frequencies that lead to the one-photon excitation of the molecule, mostly in bound (vibrational) states ($\hbox {H}_2^{*}$), whose dynamics we aim to track introducing a second pulse delayed in time. The first pulse not only excites, but can also ionize the system simultaneously, into bound and dissociative states of the ion, through the absorption of one or two photons. An illustration of the processes induced by a single pulse is given in Fig. 1b. The interaction with the first pulse creates a superposition of molecular states with components in the fundamental ($\psi _0$) and excited states of both the neutral ($\psi _m$) and the ionized molecule ($\psi _{E_f}$), which can be written as:

1 $$\begin{aligned} \Psi (t)=c_0(t)\psi _0 + \sum _m c_m(t)\psi _m + \int _{E_f} c_{E_f}(t)\psi _{E_f} \ . \end{aligned}$$

A series of simulations are then performed introducing a second identical pulse at different time delays. This second pulse thus interacts and interrogates the pumped system, described by Eq. (1), and creates the interferometric signal between the direct and the sequential two-photon paths. The resulting interference patterns will appear superimposed to the optical interferences that lead to a comb in the energy distributions as a result of using identical pulses delayed in time^25^ (see frequency distributions in supplementary information). As shown in Fig. 1b, one-photon ionization can be also energetically allowed depending on the laser parameters.

#### Interferometric ionization signal

**Figure 2:**
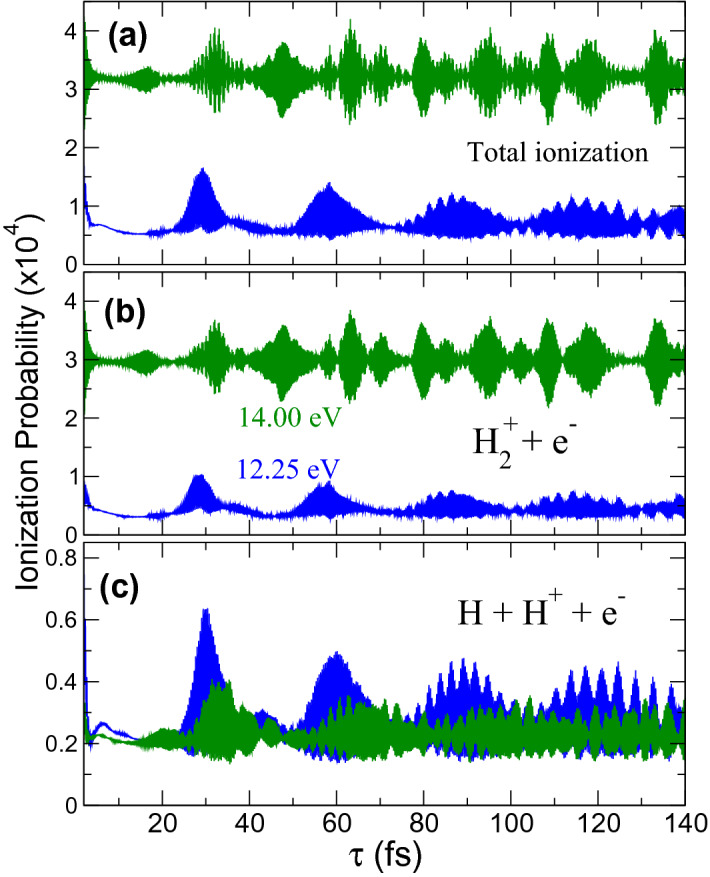
Ionization yields as a function of the time delay between pulses. Results for an scheme using 12.25 eV (blue lines) and 14.00 eV pulses (green lines). (**a**) Total ionization yield. (**b**) Non-dissociative ionization. (**c**) Dissociative ionization (note the different scales in the y-axis).

We carry out two complete sets of calculations using pulses with carrier frequencies of 12.25 eV and 14 eV. The former corresponds to a resonant transition from the ground to the first excited state of the molecule ($\hbox {B}\, ^1\Sigma _u^+$) at the internuclear equilibrium distance of $\hbox {H}_2$ (1.4 a.u.). The later leads to excitation of both the first and the second (B$'$ $^1\Sigma _u^+$) excited state. See energetics in Fig. 1b,c. Since we are using 2-fs pulses, their broad energy bandwidth populates a manifold of vibronic (electronic+vibrational) states in either scheme. Figure 2a shows the total ionization yields resulting for the 12.25 eV (blue) and the 14 eV scheme (green). We have integrated (sum) over all energetically open electronic and nuclear kinetic energy sharings, thus providing the yields that would be measured in an experiment in which only the ions or electrons were counted.

**Figure 3:**
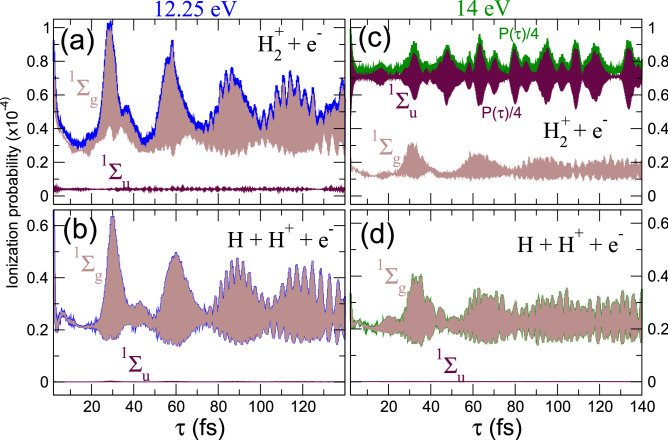
(**a**) Non-dissociative ionization probability (blue line) as a function of time for the pump-probe schemes using 12.25 eV pulses, including the contributions to a total final symmetry $^1\Sigma _g^+$ in light brown line (i.e., two-photon absorption) and $^1\Sigma _u^+$ in violet color (one-photon absorption). (**b**) Same as (**a**) for the dissociative channel. (**c**) Non-dissociative and (**d**) dissociative ionization yields for the 14 eV pulses pump-probe scheme, also including the contributions of each specific molecular symmetry.

In panels (b) and (c), we show the contributions of the non-dissociative and dissociative channels. We first observe that the probability of dissociating after ionization (Fig. 2c) is rather similar for both schemes, using 12.25 and 14 eV pulses. Furthermore, the signal exhibits somehow similar trends over time. However, the probability of ionizing the hydrogen molecule without dissociating, i.e., leading to $\hbox {H}_2^+$ (Fig. 2b) is significantly larger, almost by one order of magnitude, for the 14 eV pulses. Also, the evolution with time is quite different for the two chosen central frequencies. For 14 eV, the non-dissociative channel is the dominant contribution by far [the green line in (a) is almost identical to the green one in panel (b)]. This is because, for a pulse with a carrier frequency of 14 eV and 2 fs duration, ionization is possible by one-photon absorption. In contrast, for 12.25 eV, this transition is energetically forbidden and ionization, regardless it leads to dissociation or not, requires two photons. We can easily disentangle the contributions of one and two-photon paths by looking at specific final symmetries of the system. These contributions are plotted in Fig. 3. Notice that the contributions of different final molecular symmetries are incoherently superimposed in the angularly-integrated signal. Unless angularly-resolved photoelectron distributions are measured, paths associated to odd/even number of photons contribute to the signal but do not interfere.

We plot the dissociative and non-dissociative ionization yields for 12.25 and 14 eV pulses, with their relative contributions for a total final symmetry of the system $^1\Sigma _u^+$ or $^1\Sigma _g^+$, i.e. for an odd or an even number of absorbed photons, respectively. This figure confirms that two-photon ionization always dominates except for the non-dissociative channel when using 14 eV pulses. For 12.25 eV, the interference profiles in time are almost exactly the same, i.e. both final channels are capturing the same dynamics. However, for 14 eV pulses, non-dissociative ionization is more than four times larger than for 12.25 eV, and 10 times larger than the non-dissociative channel. Nevertheless, as we will see next, a Fourier analysis of the signal could still unveil contributions from all present channels.

Every subplot in Figs. 2 and 3 reveal two different time scales, showing a very fast oscillation with a period in the sub-femtosecond scale, and a slower oscillation with a period of the order of tens of femtoseconds. As one can expect, the first one is related to electronic motion and the second one to the nuclei. Next, we perform a detailed Fourier analysis of the signal to disentangle these time-frequency components opening the way to a quantum state holographic technique to reconstruct the molecular wave packet generated by the first (pump) pulse.

#### Time-frequency analysis

**Figure 4:**
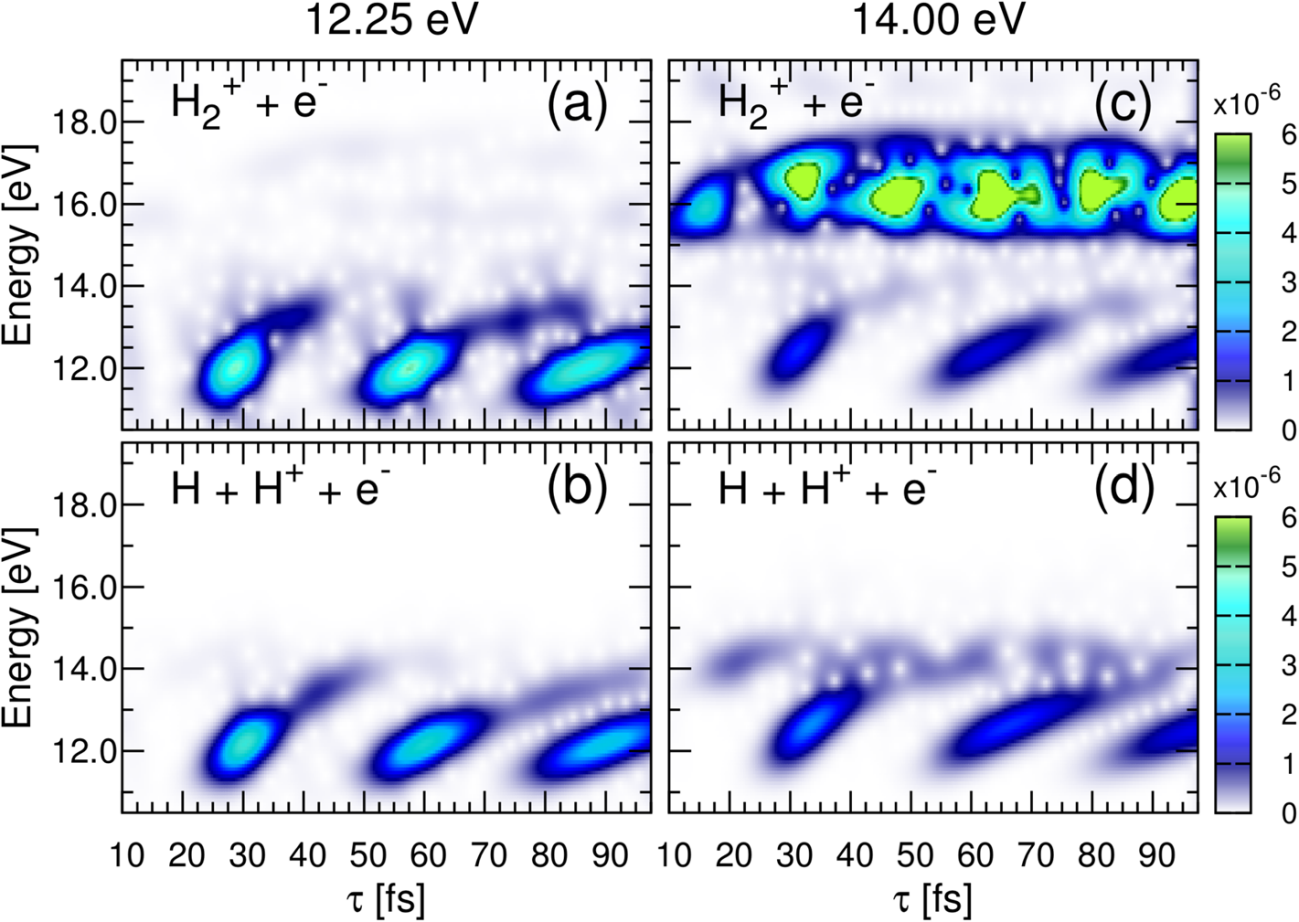
Short-Time Fourier Transform of the ionization signals given in Fig. 2. (Left) Non-dissociative and dissociative ionization probabilities as a function of energy (y-axis) and time (x-axis) for a pump-probe scheme using 12.25 eV pulses. (Right) Same for 14 eV pulses.

We perform a short-time Fourier transform (STFT) of the ionization signal employing a Gaussian window function with a 2 fs width, which give us a good compromise between time and energy resolution to disentangle the distinct time scale in the dynamics under inspection (numerical details on the STFT are given in the supplementary information). Figure 4 shows the resulting time-frequency maps of ionization yields. Left panels for 12.25 eV pulses and right panels for 14 eV pulses. We first look at dissociative ionization (panels b and d), which necessarily requires the absorption of two photons. For non-overlapping pulses, the probability of finding the system in an specific state *f* can be written for the two-photon process as^25^:

2 $$\begin{aligned} P_f^{2\omega }(\tau ) = \left| a_f \left( 1 + e^{i \Delta E_{f0}\tau }\right) + \sum _m b_{fm} e^{i \Delta E_{fm}\tau }\right| ^2 \, \end{aligned}$$

where *f* refers to a final vibronic (vibrational+electronic) state. $a_f$ is the complex amplitude corresponding to the “direct” two-photon ionization from the ground state; $b_{fm}$ is the amplitude for the sequential process, i.e., the amplitude describing excitation from the ground to the *m*-th vibronic (vibrational-electronic) state and, after a given delayed time ($\tau$), from the latter state to the ionization continuum; and $\Delta E_{f0}=(E_f-E_0)$, and $\Delta E_{fm}=(E_f-E_m)$. Note that the formula includes a coherent sum over all *m* states contributing to the wave-packet created in the intermediate states and that the energy differences $\Delta E_{f0}$ and $\Delta E_{fm}$ involve total energies, i.e., electronic plus nuclear energy, for the final state ($E_f$), the ground state ($E_0$) and all *m* intermediate states ($E_m$) of the molecule. The second term in (2), with the sum over *m*, thus carries the dynamical information of the (one-photon) excited molecule. Developing Eq. (2), we see that we will have a series of cross terms where the relative phases within the states conforming the wave packet in Eq. (1) are imprinted together with their amplitude values in $P_f(\tau )$ and, consequently, are those retrieved in the STFT shown in Fig. 4. For consistency, we keep the notation employed in our previous works^25^ and decompose the multipath signal in three main terms^13^:

3 $$\begin{aligned} P_f(\tau ) = \alpha _f(\tau ) + \beta _f(\tau ) + \gamma _f(\tau ) \end{aligned}$$

where

4 $$\begin{aligned} \alpha _f(\tau )= & {} 2|a_f|^2 \left\{ 1 + \cos [(E_f-E_0)\tau ] \right\} \nonumber \\ \beta _f(\tau )= & {} \sum _m |b_{fm}|^2 + \sum _{m,m'>m} 2{\text{ Re }}(b_{fm}b_{fm'}^*)\cos [(E_{m'}-E_m)\tau ] \nonumber \\ \gamma _f(\tau )= & {} \sum _m 2 {\text {Re}} (a_f^* b_{fm}) \left\{ \cos [(E_f-E_m)\tau ] + \cos [(E_m-E_0)\tau ] \right\} \end{aligned}$$

where, in brief, the $\alpha$ term holds the “direct” processes from the ground state, the $\beta$ term stands for the “sequential” transition in a direct map of the excited dynamics, and the $\gamma$ contribution captures the interference among them. The phase differences involving $E_f$ will be mostly washed out in the integrated signal we are looking at, i.e. the cross terms oscillating with $\cos [(E_f-E_0)\tau ]$ and $\cos [(E_f-E_m)\tau ]$ will give an almost constant value as a function of $\tau$. Consequently, only the excited dynamics, associated to those terms involving ground and intermediate states, $\cos [(E_{m'}-E_m)\tau ]$ and $\cos [(E_m-E_0)\tau ]$, will still survive and can then be fully characterized in a Fourier analysis as we discuss below. Notice that the form of Eqs. (2)–(4) is independent of the laser paremeters chosen to perform the experiment (duration, laser intensity or introducing a chirp), as long as both pulses are identical. Such modifications would only affect the values of the complex amplitudes $a_f$ and $b_{fm}$, but the interferometric signal will still oscillate with the cosine terms, as written in Eq. (4). Consequently, the absolute ionization yields would obviously vary with $a_f$ and $b_{fm}$, but the time-frequency analysis and conclussions presented next would remain unchanged when, e.g., chirped pulses will be used. Every subplot in Fig. 4 reveal features at observation energies (y-axis) given by $(E_m-E_0)$ (around 10–14 eV), i.e. fast electronic beatings that a 2-fs window can only resolve in energy and that are revealed thanks to the interferences between the two-photon direct and sequential processes ($\gamma$ term). This fast electron dynamics is coupled with the low oscillatory frequencies captured as a function of time and are given by $(E_{m'}-E_m)$, i.e. beatings between vibrational states ($\beta$ term).

For 12.25 eV pulses, we plot the non-dissociative (Fig. 4a) and dissociative ionization signal (Fig. 4b), where we clearly distinguish a compact oscillating signal repeating every $\sim 30$ fs associated to a nuclear wave packet sitting in the first excited state, B $^1\Sigma _u^+$. In Fig. 4d, for 14 eV pulses, we distinguish a second feature at observation energies 14–14.5 eV. We now significantly populate both the B and the B$'$ $^1\Sigma _u^+$ states. The STFT thus shows the trace of the slower nuclear dynamics given by vibrational states in the same or energetically close (B and B$'$ $\Sigma _u$ electronic states). Therefore, it shows the slow frequencies for the wave packet components mostly associated to the B state (around 12 eV and moving with a  30 fs periods), the faster nuclear components on the B$'$ state (around 14 eV and moving with the slower period of lower vibrational states in the higher electronic state) and the cross terms between them. See the potential energy curves of the B and B$'$ (first and second) excited electronic states of $\hbox {H}_2$ plotted in Fig. 1b.

The Fourier analysis of the non-dissociation ionization yield at 12.25 eV is identical to that of the dissociative ionization, capturing the same dynamical information. However, for 14 eV pulses, more complex profiles arise superimposed to the slightly fainter signal of the coupled nuclear motion associated to the B and B$'$ states that was already visible in the dissociative channel. The more intense trace in this time-frequency map appears at around 15.5 eV, which corresponds to the direct vertical transition from the ground state of $\hbox {H}_2$ into the lowest ionization threshold, i.e. the one-photon ionization contribution, capturing in this case with a much larger amplitude the term governed by $cos[(E_f-E_0)\tau ]$, which is energetically forbidden for 12.25 eV pulses. The probability thus incorporates the first order term:

5 $$\begin{aligned} P_f(\tau ) = P_f^{2\omega }(\tau ) + P_f^{1\omega }(\tau ) \, \end{aligned}$$

where $P_f^{2\omega }(\tau )$ was given in (2) and the second term only carries optical interferences, $P_f^{1\omega }(\tau ) = \left| a^{1\omega }_f \left( 1 + e^{i \Delta E_{f0}\tau }\right) \right| ^2$, where $a^{1\omega }_f$ is the one-photon amplitude from the ground to the final ionic state *f*. In summary, the time-frequency analysis of the total ionization signal allows us to unveil the three main contributions to the multipath interference and asses the distinct time scales associated to electrons and nuclei.

#### Direct versus sequential processes

**Figure 5:**
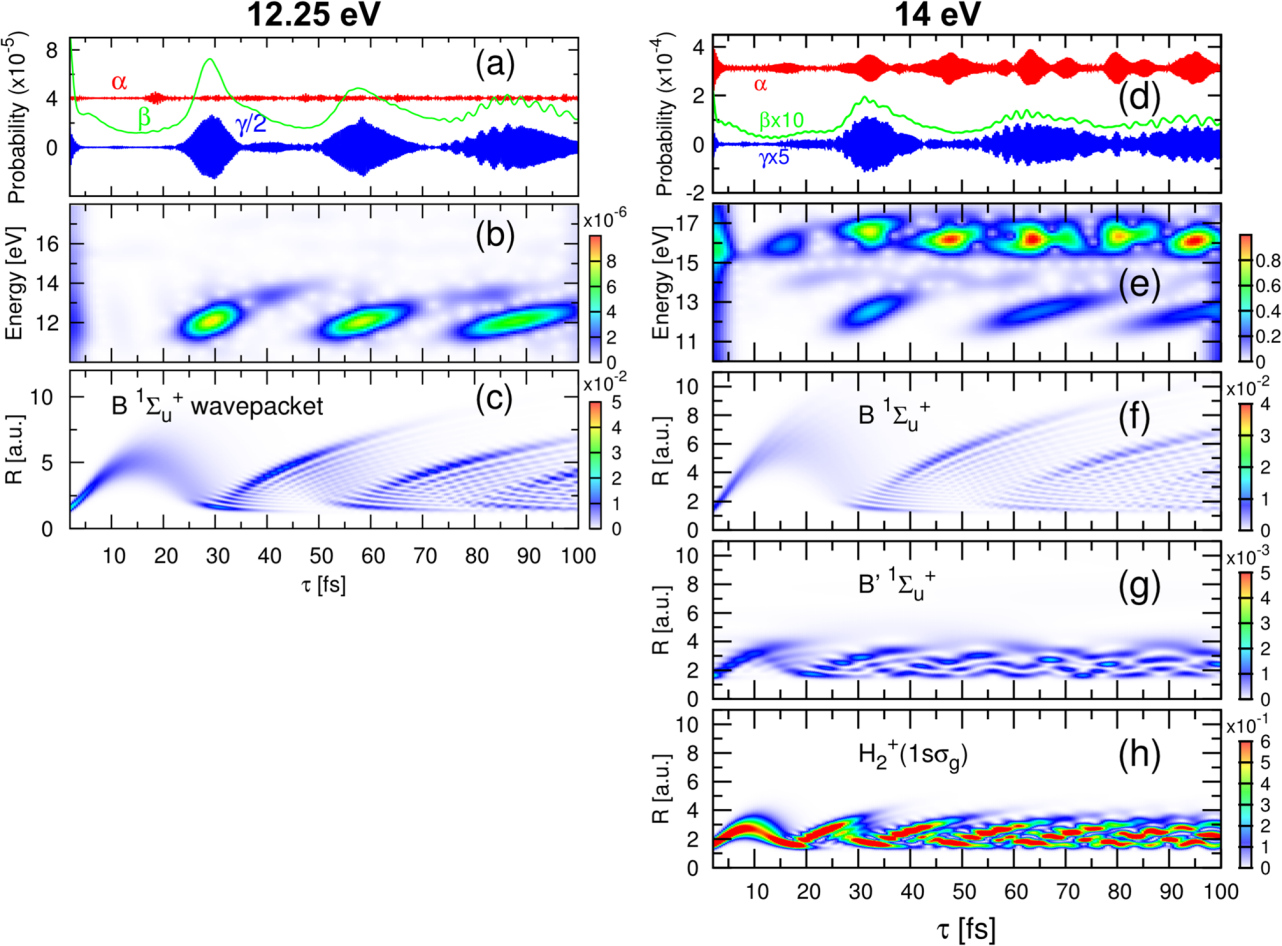
(**a**)–(**c**) 12.25 eV pulses scheme. (**d**)–(**h**) 14 eV pulses scheme. (**a**) Contributions to the ionization yield from each term defined in Eq. (4) after integration over all final states *f* for 12.25 eV pulses. (**b**) STFT of the total yield. (**c**) Nuclear wave packet distribution as a function of time resulting after the interaction with a single (pump) pulse associated to the first excited state, B $^1\Sigma _u^+$. Negligible populations are found in higher excited and ionic states for 12.25 eV pulses. (**d**) Same as (**a**) for 14 eV pulses. (**e**) STFT of the total yield. (**f**) Nuclear wave packet (NWP) distribution as a function of time resulting after the interaction with the pump pulse associated to the first excited state, B $^1\Sigma _u^+$. (**g**) NWP in the second excited state, B$'$ $^1\Sigma _u^+$. (h) NWP in the ground state of the ion, $\hbox {H}_2^+$($1s\sigma _g$).

Theory allows us to analyze in more depth of the interferogram, since we can extract separately the terms in the decomposition given in Eqs. (3) and (4)^25^, which are integrated over all final states, $E_f$. Figure 5a shows the contribution of each term to the total ionization yield for the 12.25 eV pulses scheme. As previously explained, the $\alpha$ term holds the “direct” processes from the ground state, the $\beta$ term stands for the “sequential” transition in a direct map of the excited dynamics, and the $\gamma$ contribution captures the interference among them. Figure 5a confirms that the slow oscillation (period of $\sim 30$ fs) is associated to the ”sequential” process, i.e. the $\beta$ term that solely contains a direct interrogation path of the time evolution associated to the *m* intermediate states and dictated by the $b_m$ amplitudes. The subfemtosecond oscillation appears in the $\gamma$ term, i.e. in the interference resulting from the two-photon direct and the two-photon sequential paths. The large oscillation in the probability in the $\beta$ and $\gamma$ terms with respect to the rather small variations of $\alpha$ in Fig. 5a explains the sharper features appearing in the time-frequency maps, which, as above discussed, mostly capture the NWP moving in the B state. This statement is confirmed in panel (c), where the NWP distribution of the excited molecule in the B state is plotted as a function of internuclear distance (y-axis) and time (x-axis). We see how the wavepacket moves up to internuclear distances of 4-6 a.u., reaching the classical outer turning point, and comes back to the inner side of the curve in around 30 fs. The oscillation repeats with a similar periodicity, although with the expected dephasing resulting from the frequency spreading due to the anharmonicity of the potential energy curve of the B electronic state. Panel (b) in Fig. 5 corresponds to the time-frequency analysis for the total yield, i.e. the sum of probabilities given in panels (a) and (b) in Fig. 4. We see that the wave packet spreading is nicely captured in the Fourier analysis, streatching the frequency components in time. The nice correspondence between panels (b) and (c) shows how the time-frequency map of the total ionization yield, with no specific channel extraction and integrated over all available energies, already provides a direct image of the pumped wave packet.

The same analysis is now carried out for the 14-eV pulses scheme shown in Fig. 5d–h. Despite the complex patterns introduced by the appearance of one- and two-photon paths, it is still possible to reconstruct the individual nuclear components of the excited wave packet. Figure 5d show the probability of each term of Eq. (4) and the STFT for the total ionization probability is plotted in (e) (again, sum of panels (c) and (d) in Fig. 4). We see the notable dominance of the direct one-photon ionization process in the $\alpha$ term, also captured in the time-frequency map in (e), where the components of the pumped wave packet associated to the B and B$'$ states are still distinguishable. The time profiles of the $\beta$ and $\gamma$ terms reproduce now the existence of nuclear wavepackets with different periods. We can elucidate the one-to-one correspondence of the components in the pumped attosecond molecular wave packet in the interferogram in Fig. 5e: the NWP in the B state (whose spatial distribution is given in Fig. 5f) is imprinted in the Fourier signal in (e) at frequencies around 12 eV; the faint trace of the nuclear components of the B$'$ state (NWP distributions plotted in Fig. 5g) appear around 14 eV in (e). The direct one-photon transition associated to the NWP that evolves in the fundamental state of the $\hbox {H}_2^+$ ion is captured at frequencies $\sim 16$ eV. It is thus demonstrated that even in the scenario where one- and two-photon processes contribute to ionization and no fragmentation channels are distinguishable, it is possible to retrieve the absolute phase, for every component. In the next section, we present the procedure in order to accurately extract both, phases and amplitudes, associated to the pumped wave packet from an energy differential interferogram, without requiring any prior knowledge of the molecular system.

### Quantum state holography

**Figure 6:**
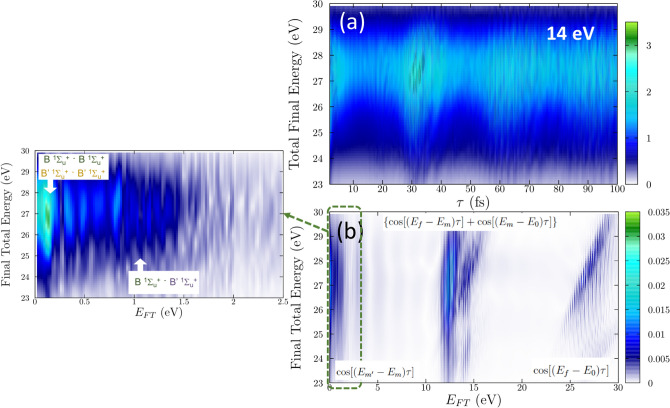
(**a**) Ionization probability as a function of total final energy, $E_f$, and time delay in a pump-probe scheme using 14 eV pulses. (**b**) Fourier Transform of (**a**). On the left, we include a zoom of this Fourier transform signal in the low-frequency range.

In this section, we show how the attosecond molecular wave packet created by the pump pulse can be reconstructred by measuring the energy differential ionization probabilities. In Fig. 6a, the ionization probabilities, $P_f(t)$ are now plotted as a function of the energy of the final molecular state, $E_f$ and as function of the time-delay between pulses. For each final energy, $E_f$ there is an infinity number of possible energy sharings between electrons and nuclei. The probabilities have then been integrated (sum) over energy sharing, but still plotted for specific final energies $E_f$ of the whole system. We demonstrate the phase-amplitude retrieval approach for the more complex scenario associated to the scheme employing 14 eV pulses, where a richer molecular dynamics is in play. In Fig. 6a, we first observe that the maximum probability oscillates in the energy range  25–29 eV, which simply reflects the broad energy bandwidth of the pulse reaching a wide range of final energies after two-photon absorption. There is a rapidly oscillating signal as a function of time that, as we have already seen, are associated to electronic beatings within the pumped wave packet.

The corresponding Fourier Transform is shown in Fig. 6b. On the y-axis we keep the absolute energy of our final state and on the x-axis the observation energy of the Fourier transform. We mostly identify in the Fourier Transform vertical lines with some diagonal patterns. At very low frequencies (0–2 eV), the beatings between energetically close vibronic states within the pumped wave packet, i.e. $(E_{m'}-E_m)$. Between 10-15 eV, we see vertical lines, i.e. a signal independent of the final energy, i.e. $(E_m-E_0)$ beatings, superimposed to the trace of a $E_f$-varying pattern, $(E_f-E_m)$ ($\gamma$ term). The higher frequency components, between 25–30 eV, correspond to the direct two-photon paths, ($E_f-E_0$)($\alpha$ term). The accumulated phase difference between the quantum paths imprinted in the $\beta$ and $\alpha$ terms, with relative phases $(E_{m'}-E_m)$ and $(E_m-E_0)$, are independent on the observation energy, $E_{FT}$, therefore they appear as vertical lines. In contrast, the Fourier frequencies of the direct and direct-sequential interferences, $(E_f-E_0)$ and $(E_f-E_m)$, increases with $E_{FT}$, since the accumulated phase difference between the direct and indirect ionization pathways is proportional to it. This results in those diagonal patterns.

**Figure 7:**
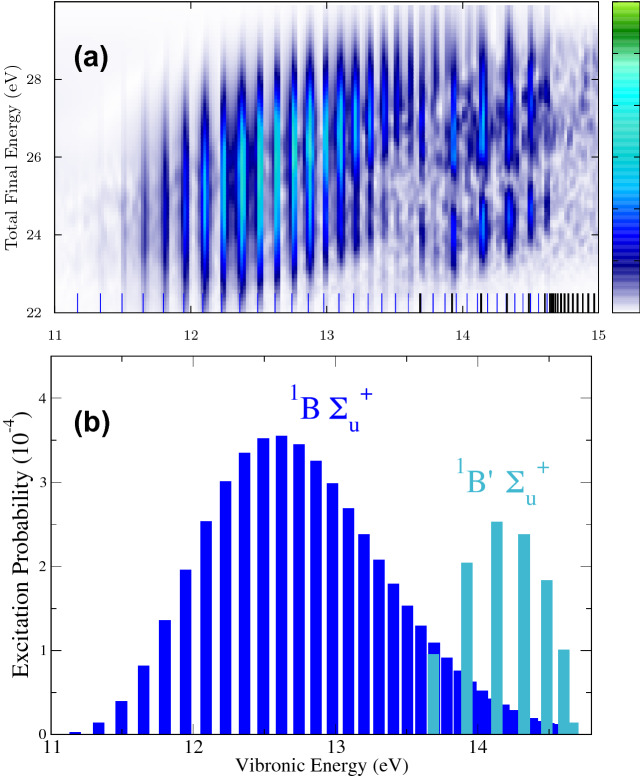
(**a**) Zoom of the Fourier transform signal of Fig. 6b in the frequency range [11–15] eV. (**b**) Excitation probabilities into the vibronic states associated to the B and B$'$ $^1\Sigma _u^+$ states, equivalently, the absolute value squared of the amplitudes for every single component that builds the wave packet under interrogation.

For a close inspection, we enlarge the interferogram in the energy regions between (0–2.5) eV in Fig. 6 (zoom) and 11–15 eV in Fig. 7a. In Fig. 6 (zoom), we observe that the low frequency components actually appear as two distinguishable manifold of frequencies: below  0.5 eV, corresponding to energy differences between vibrational states associated to the same excited state (B or B$'$), and around 1 eV that actually correspond to the beatings between vibrational states from different excited electronic states. Note that the intensity of these lines is a direct measure of the product of amplitudes $a_f*b_{fm}$ described in the $\alpha _f(\tau )$ term in Eq. (4). Complementary information on the pumped wave packet can be retrieved from Fig. 7a. We see a map revealing well defined vertical lines resulting from the interference between the direct (from the ground state of energy $E_0$) and sequential process (absorption of a second photon from the probe pulse and bringing the components *m* in the evolving pumped wave packet into the final state). We are thus uncovering the beating pattern of the cross term in the $\beta$ component in Eq. (4), $\sum _m 2 {\text{ Re }}(a_f^*b_{fm})\cos [(E_m-E_0)\tau ]$. We can observe the signature of the vibrational progressions of the B and B$'$ excited electronic states that conform the pumped wave packet in the excited molecule. More importantly, because the amplitude $a_f$ (ground to final state) remains unchanged with time delay, the intensity of the signal is directly providing a direct capture of the amplitude value corresponding to $b_{fm}$, the amplitudes for each individual *m* component. This is demonstrated by comparing the relative signals with the actual excitation probabilities after the interaction of a single 2-fs pulse centered at 14 eV, i.e. $P(E_m) = |c_m|^2$ for each intermediate state that are plotted in Fig. 7b. In other words, assuming the use of well-characterized attosecond pulses, we can accurately retrieve the relative phases and amplitudes of every component within the wave packet.

Although we have demonstrated the reconstruction procedure for linearly polarized light parallel to the molecular axis, the exact same procedure can be applied for an experiment performed with randomly oriented molecules, as long as one takes into account every optically allowed excited state. In the present pump-probe scheme for $\hbox {H}_2$, it would imply to account for two additional singly excited states of $\Pi _u$ symmetry, whose associated vibrational periods are quite different from those of the $\Sigma _u$ states considered in this work^35^. In principle, the same strategy can be applied to larger molecular targets, although the complexity of the reconstruction procedure will depend on the dynamical properties of the states that conform the pumped wave packet. Full dimensional simulations at the same level of accuracy presented in this work are not available yet for molecules much larger than $\hbox {H}_2$, although they are expected to be achievable in a near future^37^.

## Discussion

We have employed a XUV-XUV pump-probe scheme to obtain a clean map of the ultrafast dynamics triggered in excited molecules by measuring total photoion or photoelectron yields. In our previous theoretical studies^25,26^, we showed how to map the excitation dynamics into the two-photon induced dissociative ionization channel or in the angularly resolved photoelectron spectrum. However, from the experimental side, such measurements would require advanced momentum-coincidence techniques and high repetition rates that are hard to achieve with current laser technology. Therefore, we have investigated here protocols that would avoid these experimental difficulties at the expense of more complex patterns being retrieved in the total yields. In the present scheme, one- and two-photon absorption processes can simultaneously ionize the molecule through different paths that interfere among them. From the resulting interferogram, it is still possible to retrieve the information encoded in a specific path, in the two-photon sequential process, which allows us for a direct map of the pumped dynamics. We have used a quantum state holographic approach, as employed in XUV-pump/IR-probe experiments in Helium^12,15^, that allows for a full reconstruction of the pumped molecular wave packet in excited states of the target.

## Methods

The ab initio method has been described in detail elsewhere^38^. Here, we only reproduce the key steps to illustrate the formalism. For the description of the laser-molecule interaction, we numerically solve the time-dependent Schrödinger equation (TDSE) in a basis set of Born-Oppeheimer molecular eigenstates resulting of the molecular hamiltonian:

6 $$\begin{aligned} \hat{{\mathscr {H}}}^0 ( \mathbf{r} ; R) = {\hat{T}}_N (R) + \hat{{\mathscr {H}}}_{el} ( \mathbf{r} ;R), \end{aligned}$$

where ${\hat{T}}_N(R)=-\frac{1}{2\mu }\nabla ^2_R$, is the nuclear kinetic energy operator and

7 $$\begin{aligned} \hat{{\mathscr {H}}}_{el}=\sum ^{n}_{i=1} \left[ -\frac{\nabla ^2_{ i}}{2} - \frac{Z_A}{|{\mathbf {r}}_i - {\mathbf {R}}_A|} - \frac{Z_B}{|{\mathbf {r}}_i -{\mathbf {R}}_B|} \right] + \frac{(Z_A Z_B)}{R} \end{aligned}$$

is the electronic Hamiltonian, which parametrically depends on the nuclear coordinate *R*. As in prevous work, we have ignored the rotational degrees of freedom, which is reasonable for the time-scales under inspection, in the femtosecond to sub-femtosecond scale. In order to avoid the diagonalization of the total hamiltonian $\hat{{\mathscr {H}}}^0$, we employ a time-dependent Feshbach close-coupling formalism that allows us to project the total molecular wave function onto two subspaces that hold the non-resonant scattering-like and bound-like states, respectively. This formalism provides an accurate representation of the autoionizing states lying above the ionization threshold^38^. The non-resonant electronic continuum components are built as antisymmetrized products of $\hbox {H}_2^+$ states and one-electron continuum wave functions obtained as an L$^2$ close-coupling solution of the scattering problem. For the photon energy range under investigation, the electronic continua associated to the two lowest ionization thresholds, i.e. the 1s$\sigma _{\text {g}}$ and 2p$\sigma _{\text {u}}$ states of the ion, are included and combined with all possible photoelectron angular momentum up to $l^{\alpha }_{max}=7$ for the two optically allowed molecular symmetries, $^1\Sigma _{\text {u}}^+$ (odd number of absorbed photons) and $^1\Sigma _{\text {g}}^+$ (even number of absorbed photons). The one-electron functions are represented as products of spherical harmonics and B-spline functions to describe the angular and radial electronic components, respectively. Numerically converged results required a radial basis covering up to 60 a.u. and maximum angular momentum of $\hbox {l}_{max}$=11 for the electronic components of the underlying $\hbox {H}_2^+$ basis sets, and a radial box of 14 a.u. for the nuclei. Because we are working in a basis set of vibronic eigenstates, the time-dependent coefficients of our expansion directly give the probability of finding the system in a given state, and therefore, the total ionization probability retrieved for each pair of pulses can be written as:

8 $$\begin{aligned} \begin{aligned} P(\tau )=\sum _{\alpha }\sum _{l_{\alpha }}{{{\sum \!\!\!\!\!\!\!\int }\,}}_{v_{\alpha }}\int _{\epsilon }|c_{\alpha ,l_{\alpha }}(v_{\alpha },\epsilon ;T+\tau )|^{2}dv_{\alpha }d\epsilon \end{aligned} \end{aligned}$$

where $\alpha$ indicates each ionic state ($1s\sigma _{g}$ and $2p\sigma _{u}$), $v_{\alpha }$ the bound or continuum vibrational states of the residual ion, $\epsilon$ and $l_{\alpha }$ the energy and the angular momentum respectively of the ejected electron, *T* is the pulse duration and $\tau$ the time delay. For the shortest time delays, when both pulses overlap, we solve the TDSE numerically. In order to decrease the computational cost of numerically integrating the TDSE, in the elapsed time between pulses when the electric field is strictly zero, the time evolution of the molecular wave packet created after the pump pulse is carried out analytically, i.e. by simply evolving each component of the wave packet with their corresponding stationary phase, as in^13,25^.

## Supplementary information

Supplementary Information.
