# Supplementary material for: Quantum state holography to reconstruct the molecular wave packet using an attosecond XUV–XUV pump-probe technique
Source: Sci Rep. 2020 Jul 31;10:12981. doi: 10.1038/s41598-020-69733-1 (PMC7395139; doi:10.1038/s41598-020-69733-1)
Supplement: Supplementary file 1 — Supplementary Information. [file 41598_2020_69733_MOESM1_ESM.pdf]

Supplementary information: Quantum state holography to reconstruct the molecular wave packet using an attosecond XUV-XUV pump-probe technique

Alberto González-Castrillo[1], Fernando Martín[1,2,3], and Alicia Palacios[2,4,*]

[1]Instituto Madrileño de Estudios Avanzados (IMDEA) en Nanociencia, 28049 Madrid, Spain
[2]Departamento de Química, Modulo 13, Universidad Autónoma de Madrid, 28049 Madrid, SPAIN
[3]Condensed Matter Physics Center (IFIMAC), Universidad Autónoma de Madrid, 28049 Madrid, Spain
[4]Institute of Advanced Research in Chemical Sciences (IAdChem), Universidad Autónoma de Madrid, 28049 Madrid, Spain
[*]alicia.palacios@uam.es

Optical interferences

The electromagnetic field corresponding to the combined action of two identical XUV pulses, $E(t) = E_1(t) + E_2(t)$, is plotted in the left panels of figure S1 as a function of time. As common practice[1], to avoid ambiguity in the definition of the field, we first define the vector potential, $\mathbf{A}(t)$, and derive the electric field, $\mathbf{E}(t) = -\partial \mathbf{A}(t)/\partial t$. The pulses depicted in the figure are defined from a vector potential, $\mathbf{A}(t) = A(t)\varepsilon_p$ where ε_p is the polarization vector. We define $A(t) = A_0 f(t)\sin(\omega_0 t + \Phi)$ with $\omega_0 = 12.25$ eV, an initial phase $\Phi = 0$ and a cosine squared envelop function, $f(t)$, with a total duration in time of 2 fs. We are employing Fourier Transform limited pulses, i.e., we chose a constant carrier frequency in time, ω_0, which can be achievable nowadays for attosecond XUV pulses[2]. Schemes using five different time delays are included, $\tau = 0.1, 0.5, 1, 2$ and 4 fs, as accordingly labeled in each subplot. For reference, we include a common grey tick line in every panel, corresponding to a single pulse with the same parameters. The right panels in the figure show the corresponding Fourier Transforms ($|E(\omega)|$), i.e. the energy components of the combined fields. For a meaningful comparison in peak intensity, the grey full line in the right panels now corresponds to the Fourier Transform of both pulses with zero delay. These frequency representation of the pulses show the optical interferences that are imprinted in the energy differential excitation/ionization yields[3] but, which, obviously, are unrelated to any electron or nuclear dynamical effects.

Time-frequency analysis

We perform a short-time Fourier transform (STFT) of the ionization probability signal as a function of time t by using the expression:

$$F_{STFT}[f(t)](w;t_0,\sigma) = \int_{-\infty}^{\infty} f(t) g\left(\frac{(t-t_0)}{\sigma}\right) e^{(-i\omega t)} dt \qquad (1)$$

where, $F_{STFT}[f(t)](w;t_0,\sigma)$ is the STFT of the function $f(t)$, $g((t-t_0)/\sigma)$ is the window (temporal shape) function, t_0 is the time center point of the window function and σ is the bandwidth in time of the window function. As commonly employed, we use a Gaussian-shape function to simplify the numerics:

$$g\left(\frac{(t-t_0)}{\sigma}\right) = \frac{1}{\sqrt{2\pi}\sigma} e^{\left(-\frac{(t-t_0)^2}{2\sigma^2}\right)} \qquad (2)$$

where the factor $\frac{1}{\sqrt{2\pi}\sigma}$ is the normalization constant. First, we employ different widths for the Gaussian window function in order to elucidate an optimal width for the time-frequency analysis of our signal. We first take the dissociative ionization signal corresponding to the 14 eV pulses [green line in figure 2(c) of the main manuscript], which is mostly dominated by two-photon absorption as discussed in the main manuscript. The resulting time-frequency maps are plotted in figure S2 for

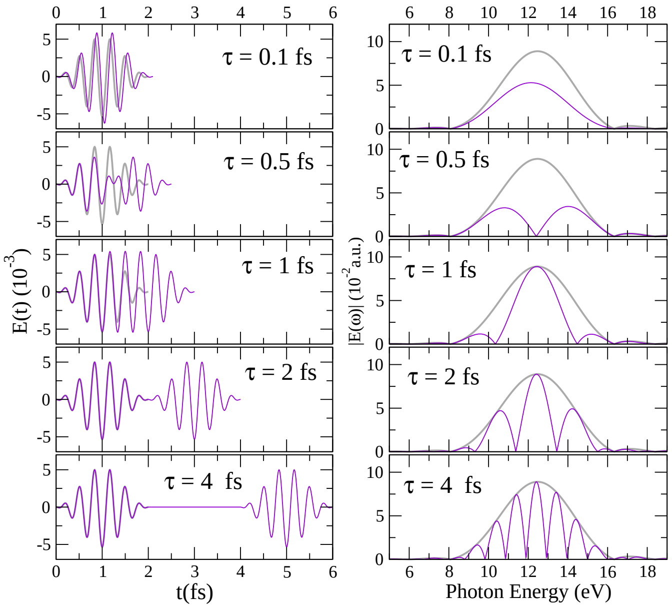

Figure S1. Electronic field corresponding to a pump-probe scheme using two identical pulses, $E(t)$ and $E(t-\tau)$ for different time delays τ. Left panels show the electric field as a function of time, while the right panels show the corresponding absolute value of the electric field as a function of frequency, i.e. the Fourier transform of the field $E(t)$ on the left side. The pulse parameters of the pulses are a total duration $T = 2$ fs, a central frequency $\omega_0 = 12.25$ eV and a peak laser intensity of $I = 10^{12}$ W/cm^2. For reference, we include on the left panels the electric field in time for a single pulse and on the right panels the frequency distribution for a pump-probe scheme with zero delay.

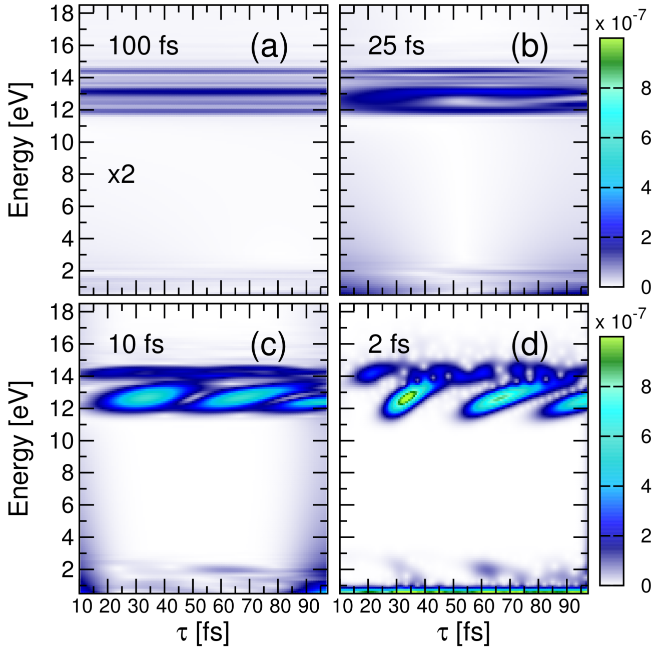

Figure S2. Short-time Fourier Transform (STFT) of the dissociative ionization signal for a pump-probe scheme with 14 eV pulses plotted in figure 2 of the main manuscript, green line in the bottom panel. Each panel correspond to the STFT performed with a different time window as described in the text: (a) 100 fs, (b) 25 fs, (c) 10 fs and (d) 2 fs.

widths of (a) 100 , (b) 25, (c) 10 and (d) 2 fs. In Fig. S2(a), we plot the outcome of a short-time Fourier transform using the 100 fs window. We can see that it is equivalent to having performed a standard Fourier Transform, i.e. we have a high spectral resolution in frequency, but not enough time resolution to disentangle processes occurring at different time scales. The observation energies (y-axis), i.e. the energy values at which well-defined lines appear in the figure, retrieved in the STFT shown in Fig. S2(a) correspond to energy beatings resulting in the cross terms defined in equations (4) of the main manuscript. This STFT is capturing the low frequency components of the intermediate wave packet associated to the energy beatings between vibrational states associated to the same or energetically close (B and B' Σ_u) electronic states, appearing between 0 and 2 eV, i.e. $(E_{m'} - E_m)$. The potential energy curves of the B and B' (first and second) excited electronic states of H_2 were shown in Fig. 1(c) in the main manuscript, together with an illustration of the coupled nuclear wave packets that are pumped in those. In the STFT in Fig. S2(a), we also distinguish two sets of higher frequencies: one in the energy range between 12 and 13.5 eV and a second one between 14 and 14.5 eV, corresponding to the $(E_m - E_0)$ cross terms associated to transitions from the ground into the B or the B' $^1\Sigma_u^+$ states. Because there are two main electronic states B and B' populated, we capture the vibrational progressions of these states.

For a 25-fs window, Fig. S2(b), we observe the appearance of a temporal variation that is more clear in the low frequency region, revealing a slow dynamics associated to the vibrational motion in the excited molecule, terms $E_{m'} - E_m$. The time-frequency analysis with smaller windows further allows us to unveil the time scales associated to each frequency manifold. Maps (c) and (d), i.e. time windows of 10 and 2 fs, respectively, already capture well distinct time scales of wave packet components at observations energies between 12 and 15 eV, i.e. the nuclear wave packets (NWPs) sitting in the B and B' excited states. The periodicity of the beat signal carries information on the nuclear components associated to each electronic state and, as expected, is given by vibrational energy gaps between m states that are very close in energy, even though it they belong to different electronic states. The absolute values of time where the maxima and minima appear will depend on the relative phases associated to those states $(E_m - E_0)$. At frequencies around $\sim$12.25 eV, we see the clear signature of a nuclear wave packet (NWP) oscillating with a period around 30 fs, corresponding to an average value of the period of the vibrational manifold generated in the first excited state that is vertically reached at that energy. As expected, the signal is thus always localized at the same frequency components, i.e. the population of these states remains unaltered in time, although we observe features that spread in the time domain as a consequence of nuclear dephasing introduced by a quite anharmonic potential energy curve (see Fig. 1(c) in main manuscript). A similar behaviour is found in the frequencies placed around 14.5 eV, energy corresponding to the vertical transition from the ground to the B' state, with a faster nuclear motion because lower vibrational frequencies are populated in this higher lying excited electronic state. We can then conclude that the 2-fs window gives us a good compromise between time and energy resolution to disentangle the distinct time scale in the dynamics under inspection. We have thus picked a 2-fs time window to perform the Fourier analysis in every measurable ionization observable, i.e. the ionization yields shown in figures 4 and 7 in the main manuscript.

References

1. Palacios, A., Sanz-Vicario, J. L. & Martín, F. Theoretical methods for attosecond electron and nuclear dynamics: applications to the H_2 molecule. J. Phys. B: At. Mol. Opt. Phys. **48**, 242001, DOI: 10.1088/0953-4075/48/24/242001 (2015).
2. Fabris, D. et al. Synchronized pulses generated at 20 eV and 90 eV for attosecond pump-probe experiments. Nat. Photonics **9**, 383–387, DOI: 10.1038/nphoton.2015.77 (2015).
3. Palacios, A., Gonzalez-Castrillo, A. & Martin, F. Molecular interferometer to decode attosecond electron-nuclear dynamics. Proc. Natl. Acad. Sci. **111**, 3973–3978, DOI: 10.1073/pnas.1316762111 (2014).
